# Occupational Therapy for People Living With Human T-Lymphotropic Virus 1: Importance and Effectiveness of an Intervention Plan at the Level of Prevention and Rehabilitation

**DOI:** 10.3389/fmed.2022.859889

**Published:** 2022-03-29

**Authors:** Edilson Coelho Sampaio, Cintia Yolette Urbano Pauxis Aben-Athar, Denise da Silva Pinto, Antonio Carlos Rosário Vallinoto, Izaura Maria Vieira Cayres Vallinoto

**Affiliations:** ^1^Institute of Health Sciences, Federal University of Pará, Belém, Brazil; ^2^Laboratory of Virology, Institute of Biological Sciences, Federal University of Pará, Belém, Brazil; ^3^Laboratory of Studies in Functional Rehabilitation of the Tropical Medicine Center, Federal University of Pará, Belém, Brazil

**Keywords:** HTLV-1, HAM, activity of daily living, occupational roles, intervention plan, occupational therapy, public health policy

## Abstract

**Introduction:**

Human T-lymphotropic virus 1 (HTLV-1)-associated myelopathy (HAM) restricts activities of daily living (ADLs), affecting health and quality of life. Occupational therapy is used to promote independence during ADL in people living with HTLV (PLHTLV).

**Objective:**

To quantify the clinical aspects, levels of functionality, performance in ADLs and occupational roles of PLHTLV and propose an occupational therapeutic intervention.

**Method:**

A cross-sectional, descriptive, observational study was designed with 40 PLHTLV monitored at two referral laboratories of the Federal University of Pará. The Evandro Chagas Research Institute Neurological Disability Scale (EIPEC-2), the Barthel Index and the Occupational Roles Identification List were applied. The G test and Fisher’s exact test (to identify associations between qualitative variables), the Mann–Whitney test (to identify associations between quantitative variables) and Pearson correlation analysis (to identify associations between the total Barthel Index and EIPEC-2 scores) were performed with Microsoft Excel and BioEstat 5.0, and the significance level was set at *p* ≤ 0.05.

**Results:**

Motor aspects, the presence of spasticity, sensory aspects, and pain in the lumbar region and lower limbs were significantly (*p* = 0.0002) higher among symptomatic individuals, who also had more difficulties urinating, traveling up and down stairs and transferring from a chair to a bed. Being a worker, performing housework and socializing (with friends or family members) were the most affected activities among HAM patients.

**Conclusion:**

The impact of HAM on PLHTLV should include an intervention plan with occupational therapists in rehabilitation programs to create an important third-level prevention initiative, which may help achieve short-, medium-, and long-term goals. Asymptomatic PLHTLV should also be able to prevent future ADL impairment.

## Introduction

Human T-lymphotropic virus (HTLV) belongs to the family Retroviridae, subfamily Orthoretrovirinae, and genus *Deltaretrovirus* and was isolated for the first time in the United States in 1980 (1). HTLV-1 is distributed globally. Based on available data, HTLV-1 is considered an ancient infection, with approximately 20 million people infected worldwide (2, 3). However, due to the lack of information on the prevalence of the infection, this number is underestimated, i.e., the actual number of infected individuals may be higher (2–6).

In Brazil, HTLV-1 infection is considered endemic, and all Brazilian regions have reported HTLV-1 and HTLV-2 infections; however, the prevalence rates differ from one state to another and are highest in the states of Bahia, Pernambuco, and Pará (7). In the state of Pará, several studies have demonstrated the endemicity of HTLV-1 in the Amazon region, with prevalence rates ranging from 0.2 to 5.3% (8). The main groups analyzed were blood donors, people with HIV-1, Japanese immigrants and quilombola and indigenous populations (9–12). The transmission of HTLV can occur through blood and blood product transfusions and through sharing needles and syringes among intravenous drug users (13–15). The main forms of HTLV-1 transmission are sexual transmission, maternal-infant transmission, especially breastfeeding, and parenteral transmission (3).

Human T-lymphotropic virus-1 is associated with broad severe pathologies, such as T-cell malignancy (adult T-cell leukemia – ATL) and inflammatory disorders (uveitis, infective dermatitis, polymyositis, and bronchitis), particularly HTLV-1-associated myelopathy (HAM), which is a neurodegenerative and inflammatory disease with a slow and progressive evolution that occurs more frequently in women than men aged 40–50 years (2, 16, 17). The neurological manifestations related to HTLV-1 are characterized by a broad spectrum, i.e., the HTLV-1 neurological complex (17, 18), which can cause difficulties regarding employment and occupational roles, in addition to compromising the quality of life of people living with HTLV (PLHTLV). Accordingly, occupational therapy, as a field of knowledge based on a biopsychosocial understanding of occupations, considers that occupations are fundamental in the lives of people, highlighting their consequent therapeutic value. Occupational therapy aims to help individuals engage in significant occupations and occupational roles through interventions (19) because occupations are essential for health and well-being (20).

Occupational roles are “guidelines” for the behavior of individuals in each context, providing structure for the time required to perform personal activities and those required by society. They are representations that promote individual identity. An individual’s place in society develops through these roles (21). These occupations and occupational roles may be affected by the clinical and neurological manifestations of HAM.

Human T-lymphotropic virus 1-associated myelopathy is marked by inflammation in the spinal cord, which causes motor and sensory impairment, bladder and intestinal sphincter disorders and erectile dysfunction (17, 22–24). Some characteristics are commonly observed in symptomatic patients and manifest in the following order: mild sensory and motor signs followed by urinary disorders and subsequent weakness in the lower limbs (25).

Motor signs and symptoms in symptomatic patients are marked by changes in gait and the presence of spasticity, with involuntary contractions of flexor and extensor muscle groups, especially in the lower limbs, thus affecting functional aspects such as climbing up and down stairs, running and jumping. Conversely, sensitivity symptoms are related to the occasional or permanent appearance of paresthesia and pain in the lower back and lower limbs. Thus, the use of systematic rehabilitation programs guided by a multidisciplinary team is essential because a decline in or loss of functional capacity (FC) affects autonomy and consequently the quality of life of these patients (26). Another relevant clinical and functional aspect is the change in bladder control. Neurological changes can compromise bladder function, leading to a state of neurogenesis, causing voiding dysfunctions such as nocturia, urgency, dysuria, hesitancy and straining to urinate, feelings of incomplete emptying, and urinary incontinence (27, 28).

The appearance of signs and symptoms linked to functional losses has diverse impacts on functionality or FC. FC can be defined as the degree of preservation of the ability to perform activities of daily living (ADLs) and instrumental activities of daily living (IADLs) in an autonomous and independent manner, which require physical, mental, and environmental conditioning skills, such as overcoming physical barriers and time limitations (29).

Given the above considerations regarding the infectious agent and its specificities, the prevalence of HTLV in the North region of Brazil and the clinical, epidemiological, functional, and occupational characteristics of patients infected with HTLV-1, we analyzed the associations of clinical aspects, levels of functionality, performance in ADLs and occupational roles in groups of symptomatic HAM patients treated in a reference unit and proposed an occupational therapeutic intervention plan for symptomatic cases.

## Materials and Methods

### Type of Study

This study entailed observational, descriptive, and cross-sectional design with a quantitative approach. The study was conducted from February to October 2019 at the Laboratory of Clinical and Epidemiology of Endemic Diseases of the Tropical Medicine Center (Núcleo de Medicina Tropical – NMT) and the Laboratory of Studies in Functional Rehabilitation (Laboratório de Estudos em Reabilitação Funcional – LAERF) of the Institute of Health Sciences of the Federal University do Pará (Universidade Federal do Pará – UFPA).

### Sampling and Ethical Aspects

Sampling in the present study was based on a spontaneous demand for care from patients diagnosed with HTLV, and the sample was composed of regularly registered patients who sought the service for periodic evaluations. Contact was made with the patients on the day of the medical consultation when the objective of the study was clarified and the invitation to participate was extended. A total of 40 individuals were selected and evaluated. The selected subjects were diagnosed with HTLV-1 infection and were 18 years of age or older. All individuals who agreed to participate in the study signed an informed consent form. This study was submitted to and approved by the Research Ethics Committee of the Health Sciences Institute (Instituto de Ciências da Saúde – ICS) of the Federal University of Pará (Universidade Federal do Pará – UFPA) (opinion 1.592.979).

The study subjects were divided into two groups: (i) symptomatic patients with a clinical and laboratory diagnosis of HAM based on the Castro-Costa criteria (22) and (ii) asymptomatic patients. The instruments used for data collection included an adapted semistructured interview questionnaire (30), the Barthel Index, the Evandro Chagas Research Institute Neurological Disability Scale (EIPEC-2) (31) and the Occupational Roles Identification List. Notably, none of the analyzed patients underwent a therapeutic intervention during the study period.

### Data Analysis

The information obtained was stored and organized to build a database in Microsoft Excel 2007 with the goal of analyzing the sociodemographic, clinical, epidemiological, and functional profiles of patients infected with HTLV-1 using descriptive statistics. Inferential statistical analysis was performed using the program BioEstat 5.0. (32) to analyze clinical findings, levels of functionality, performance in ADLs, and occupational roles between groups. The G test and Fisher’s exact test were used to identify the association between qualitative variables (Barthel Index and Occupational Roles List), the Mann–Whitney test was used to identify associations between quantitative variables (EIPEC-2 scores), and Pearson correlation was used to identify the association between the total Barthel Index and EIPEC-2 scores. The significance level adopted was 5% (*p* ≤ 0.05).

## Results

The study population consisted of 27 (67.5%) asymptomatic and 13 (32.5%) symptomatic carriers. The comparative analysis of clinical and functional characteristics using the EIPEC-2 indicated a statistically significant difference (*p* = 0.0002) between the groups of patients (Table 1).

**TABLE 1:** Distribution of patients infected by HTLV-1 based on the clinical and functional characteristics of the Evandro Chagas Research Institute Neurological Disability Scale (EIPEC-2) by group of patients.

Evaluation	Asymptomatic (*n* = 27)	Symptomatic (*n* = 13)	*p*-Value
		
	Median (ID)	Median (ID)	
EIPEC-2	2.0 (4.0)	13.0 (15.0)	0.0002

*Mann–Whitney test.*

*ID, interquartile deviation.*

A higher frequency of females was observed (75%). When the clinical classification was subdivided by sex, we found female ratios of 77.8% in the asymptomatic group and 69.2% in the symptomatic group.

When considering the distribution of patients evaluated according to age group, we observed a greater number of asymptomatic individuals in the age group of 41–50 years (33.3%) and of symptomatic individuals in the age group of 51–60 years (53.8%).

Table 2 shows the most frequent signs and symptoms in the symptomatic group and whether these were present in the asymptomatic group and shows the level of functionality of both groups. In the motor score category “walking,” 26 asymptomatic patients (96.3%) had a preserved walking ability, with a score of 0 (normal). The highest score frequency in the symptomatic group was 1 (abnormal but walking independently) (*p* = 0.003). For the “running” category, most asymptomatic patients (25/92.6%) were able to run, and nine symptomatic patients (69.2%) were unable to run (*p* = 0.0001). In the category “climbing stairs,” in the symptomatic group, six patients (46.2%) scored 1 (climbing stairs only by holding the handrail), and four (30.8%) scored 2 (unable to climb stairs). Among asymptomatic patients, most (96.3%) were able to climb stairs, even without the aid of a handrail (*p* = 0.0001). The “jumping” task was performed without difficulty in 92.6% of asymptomatic patients, and 61.5% of symptomatic patients presented difficulty (score of 3, unable to jump) (*p* = 0.002).

**TABLE 2:** Distribution of patients infected by HTLV-1 based on clinical and functional characteristics and clinical classification.

Variable	Asymptomatic (*n* = 27)		Symptomatic (*n* = 13)		*p*-Value
	*n*	%	*n*	%	
**Motor score**					
** Gait**					
0. Normal	26	96.3	03	23.1	0.0030
1. Abnormal, but walks independently	01	3.7	05	38.5	
3. Abnormal and depends on permanent unilateral support	00	0.0	02	15.4	
6. Abnormal, permanent bilateral support, and occasional use of WC	00	0.0	01	7.7	
9. Permanent use of WC, standing up with help, standing with support	00	0.0	01	7.7	
1. Permanent use of WC, unable to stand up	00	0.0	01	7.7	
** Running**					
Runs	25	92.6	04	30.8	0.0001
Unable to run	02	7.4	09	69.2	
** Climbing stairs**					
Climbs up stairs	26	96.3	03	23.1	0.0001
Climbs up stairs only with handrail	01	3.7	06	46.2	
Unable to climb up stairs	00	0.0	04	30.8	
** Jumping**					
Jumps with 1 or 2 feet	25	92.6	04	30.8	0.0020
Jumps only with both feet	00	0.0	01	7.7	
Unable to jump	02	7.4	08	61.5	
**Spasticity score**					
Clonus					1.0000
Absent	26	96.3	06	46.2	
Only induced by the examiner	00	0.0	00	0.0	
Spontaneous	01	3.7	07	53.8	
**Flexor/extensor spasms**					
Absent	25	92.6	05	38.5	0.0010
Present	02	7.4	08	61.5	
**Sensitive score**					
Paresthesia					0.0001
Absent	18	66.7	02	15.4	
Present occasionally	09	33.3	04	30.8	
Present constantly	00	0.0	07	53.8	
Lumbar pain					0.0300
Absent	10	37.0	02	15.4	
Present occasionally	12	44.4	03	23.1	
Present constantly	05	18.5	08	61.5	
Lower limb pain					0.0060
Absent	14	51.9	03	23.1	
Present occasionally	12	44.4	04	30.8	
Present constantly	01	3.7	06	46.2	
**Sphincter score**					
** Bladder control**					
Total	22	81.5	04	30.8	0.0070
Urgency	04	14.8	05	38.5	
Incontinence or occasional retention	01	3.7	04	30.8	
** Fecal control**					
Normal	23	85.2	08	61.5	0.1000
Constipation	04	14.8	05	38.5	

In terms of the spasticity score, the category “flexor/extensor spasms” was significantly different (*p* = 0.001) between the groups analyzed. These signs and symptoms were more frequent in symptomatic patients (61.5%). Only nine asymptomatic patients (33.3%) occasionally exhibited symptoms in the sensitive score category “paresthesia,” and seven of the symptomatic patients (53.8%) presented this sensory change chronically (*p* = 0.0001).

In the “lumbar pain” category, pain symptoms were recorded in both groups; occasional pain was more frequent in asymptomatic patients (44.4%), and chronic pain was more frequent in symptomatic patients (61.5%) (*p* = 0.03). Patients in the symptomatic group had more frequent complaints about pain in the “lower limb pain” category, even if only occasionally (*p* = 0.006).

The sphincter score in the “bladder control” category was significantly different (*p* = 0.007) between asymptomatic and symptomatic patients. Only five asymptomatic patients (18.5%) had some sphincter change, while nine symptomatic patients (69.3%) had some change, such as urgency, incontinence or occasional retention (Table 2).

As shown in Table 3, all asymptomatic patients performed ADLs independently. Among symptomatic patients, 10 (76.9%) performed ADLs independently, and three (23.1%) were dependent (partially or completely). For ADL feeding, dressing, bathing, personal hygiene and evacuation, asymptomatic patients were independent. In contrast, symptomatic patients required help for more than one ADL and were dependent on others for at least one of the five ADLs listed above. The need for help when dressing was more frequent in the symptomatic group (61.5%).

**TABLE 3:** Distribution of patients based on the total Barthel Index score by group.

Barthel Index	Asymptomatic (*n* = 27)		Symptomatic (*n* = 13)		*p*-Value
	*N*	%	*n*	%	
Level of functional independence					0.0300
Independent	27	100.0	10	76.9	
Dependent	00	0.0	03	23.1	

Additionally, the ADLs “urination,” “chair-to-bed transfer” and “climbing up and down stairs” were significantly different between the groups (*p* = 0.006, *p* = 0.03, and *p* = 0.0004, respectively). Notably, symptomatic patients reported the need for help from others to perform these ADLs, and to a lesser extent, patients exhibited dependence in all the aforementioned ADLs.

The data related to occupational roles are recorded in Table 4. The “worker” occupational role, which included part-time or full-time (formal or informal) paid employment, was significantly different between groups (*p* = 0.04). Most asymptomatic patients (25 patients/59.3%) but only three symptomatic patients (23.1%) were “workers.”

**TABLE 4:** Distribution of patients based on the Occupational Roles Identification List score by group.

Variable	Asymptomatic (*n* = 27)		Symptomatic (*n* = 13)		*p*-Value
	*n*	%	*n*	%	
**Occupational role**					
** Student**					
PAST					0.0600
Yes	11	40.7	1	7.7	
No	16	59.3	12	92.3	
PRESENT					0.6000
Yes	02	7.4	02	15.4	
No	25	92.6	11	84.6	
FUTURE					0.3000
Yes	12	44.4	03	23.1	
No	15	55.6	10	76.9	
** Worker**					
PAST					0.6000
Yes	25	92.6	11	84.6	
No	02	7.4	02	15.4	
PRESENT					0.0400
Yes	16	59.3	03	23.1	
No	11	40.7	10	76.9	
FUTURE					0.7000
Yes	23	85.2	10	76.9	
No	04	14.8	03	23.1	
**Volunteer**					
PAST					1.0000
Yes	10	37.0	05	38.5	
No	17	63.0	08	61.5	
PRESENT					0.3000
Yes	02	7.4	02	15.4	
No	25	92.6	11	84.6	
FUTURE					0.1000
Yes	05	18.5	06	46.2	
No	22	81.5	07	53.8	
**Occupational roles**					
** Caregiver**					
PAST					0.3000
Yes	23	85.2	13	100.0	
No	04	14.8	00		
PRESENT					0.1000
Yes	20	74.1	06	46.2	
No	07	25.9	07	53.8	
FUTURE					0.4000
Yes	23	85.2	09	69.2	
No	04	14.8	04	30.8	
** Housework**					
PAST					0.2000
Yes	26	96.3	11	84.6	
No	01	3.7	02	15.4	
PRESENT					0.0006
Yes	26	96.3	06	46.2	
No	01	3.7	07	53.8	
FUTURE					0.0080
Yes	27	100.0	09	69.2	
No	00	0.0	04	30.8	
**Occupational role**					
** Friend**					
PAST					0.7000
Yes	20	74.1	11	84.6	
No	07	25.9	02	15.4	
PRESENT					0.0100
Yes	13	48.1	01	7.7	
No	14	51.9	12	92.3	
FUTURE					0.0800
Yes	24	88.9	08	61.5	
No	03	11.1	05	38.5	
** Family member**					
PAST					0.6000
Yes	22	81.5	12	92.3	
No	05	18.5	01	7.7	
PRESENT					0.0020
Yes	21	77.8	03	23.1	
No	06	22.2	10	76.9	
FUTURE					0.0900
Yes	26	96.3	10	76.9	
No	01	3.7	03	23.1	
**Occupational role**					
** Churchgoer**					
PAST					0.7000
Yes	14	51.9	08	61.5	
No	13	48.1	05	38.5	
PRESENT					1.0000
Yes	16	59.3	07	53.8	
No	11	40.7	06	46.2	
FUTURE					0.3000
Yes	25	92.6	10	76.9	
No	02	7.4	03	23.1	
** Hobby/Amateur**					
PAST					0.1000
Yes	18	66.7	12	92.3	
No	09	33.3	01	7.7	
PRESENT					0.7000
Yes	09	33.3	03	23.1	
No	18	66.7	10	76.9	
FUTURE					0.0900
Yes	26	96.3	10	76.9	
No	01	3.7	03	23.1	
** Participation in organizations**					
PAST					0.5000
Yes	02	7.4	00	0.0	
No	25	92.6	13	100.0	
PRESENT					0.1000
Yes	02	7.4	01	7.7	
No	25	92.6	12	92.3	
FUTURE					0.1000
Yes	02	7.4	01	7.7	
No	25	92.6	12	92.3	

A significant difference for the occupational role “housework” was found between the groups in the present and in the future (past/*p* = 0.2; present/*p* = 0.0006; future/*p* = 0.008). Both the occupational roles “friend” and “family members” were linked to “social participation,” with significant differences in performance between the groups (*p* = 0.01 and *p* = 0.002, respectively).

**FIGURE 1:**
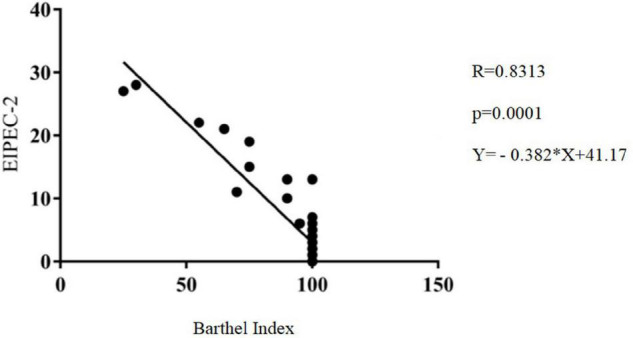
Correlation of the Evandro Chagas Research Institute Neurological Disability Scale (EIPEC-2) with the Barthel Index, Belém, Pará.

Figure 1 shows a strong correlation (*r* = 0.8313) between the two EIPEC-2 scales and the Barthel Index, indicating an association between clinical and functional variables (*p* = 0.0001).

**FIGURE 2:**
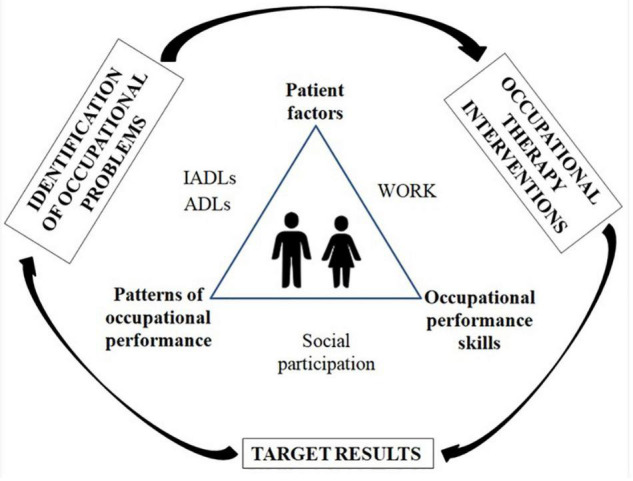
Flowchart of the proposal for an occupational therapy intervention plan for people living with HTLV-1. ADLs, activities of daily living; IADLs, instrumental activities of daily living.

From the evaluation of asymptomatic and symptomatic patients in this study and using the document “*Estrutura da Prática da Terapia Ocupacional: domínio e processo*” (“Structure of the Occupational Therapy Practice: domain and process”) as a reference (33), possible occupational problems were identified: (i) impairment of the patient (functions of the body), (ii) impairment of performance skills (motor skills), (iii) changes in performance patterns (occupational roles), and (iv) changes in performance of occupations (ADLs, IADLs, work, and social participation). Based on these findings, a proposal for an occupational therapy intervention plan (Supplementary Material and Figure 2) was developed with definitions of occupational domains, the identification of occupational problems of patients infected with HTLV-1 and HAM, interventions and target outcomes.

## Discussion

The performance of ADLs by individuals infected by HTLV-1 with HAM is impaired. In a study conducted at an HTLV reference center in Salvador (Bahia), these changes were related not only to mobility/locomotion or functional mobility (34) but also to the ability to dress oneself and self-care (35).

Accordingly, rehabilitation programs should include an occupational therapist to support the performance of ADLs and other occupations to allow PLHTLV to achieve and maintain an optimal level of functionality while interacting with their environments. Therefore, evaluations and interventions should be closer to the reality of these individuals.

Human T-lymphotropic virus 1-associated myelopathy symptoms are more frequent in women (36) and manifest predominantly in the fourth and fifth decades of life (37). The literature reports that the incidence and prevalence of all HTLV-1 neurological manifestations remain underexplored, sometimes resulting in manifestations or even oligosymptomatic forms of the disease being overlooked (17, 38).

The appearance of signs and symptoms associated with functional losses causes diverse impacts on the functionality or FC and on the performance of ADLs. In the present study, data were not obtained related to the time that the patient lived with HAM symptoms; therefore, this variable could not be associated with the performance of ADLs. However, the relationship between performance and the symptoms investigated during data collection was established using data collected through the Barthel Index and EIPEC-2 instruments. These associations are known to be relevant in terms of broadening the understanding of the limitations of patients with HAM symptoms with respect to ADLs and other occupations and warrant greater attention in HAM studies.

In the present study, symptomatic patients experienced more losses regarding occupational roles. This finding should be of interest to multidisciplinary teams and lead to the development of strategies and actions that contribute to the involvement of PLHTLV in their occupational roles and occupations in general, including the maintenance of affective relationships and support and social support networks (39), and thus combat the social exclusion of individuals with HTLV and HAM.

Among the occupational roles, housework was affected because the demands related to this role include the preservation of motor skills and factors such as body functions and structures. Participation should be supported by providing, for example, guidelines on energy conservation techniques, such as breaks in activity before the onset of fatigue (40). Changes in occupational roles can also be observed in people living with HIV or AIDS. Negative repercussions were identified in workers and in social participation (friends and family members). Prejudice and low social acceptance markedly influence the social participation of these people (41).

People affected by HTLV can also experience stigmas, whether from family members, sexual partners, friends, or even oneself. Moral implications are attributed to sexually transmitted infections (STIs), embarrassment on the part of PLHTLV and pejorative connotations about behavior considered deviant by society that depreciate infected individuals given the burden of prejudice and the lack of knowledge about STIs. In addition, infected individuals perceive disapproval from others in relation to sexual affective experiences prior to infection; feel morally judged when labeled promiscuous; and feel hurt, ashamed and guilty because of HTLV infection (42). Prejudice and discrimination may compromise the social participation of these people more so than clinical aspects.

Individuals fulfill occupational roles in their daily lives through performance standards and their habits, routines, roles, and rituals, employing performance skills in different contexts and environments (21). Individuals who suffer from chronic conditions, for example, PLHTLV, can experience interruptions or changes in occupations due to changes in body functions and structures.

Given the restrictions of occupational roles, when comparing the present to past and future expectations, these individuals must readapt and should be encouraged to participate in occupational roles that are important to them, with consideration of independence and technological maximization to help these people (43).

Therefore, these people must be assisted by a qualified multidisciplinary team within an integrated health care network that includes an occupational therapist—a professional who facilitates the therapeutic use of occupations in individuals or in groups—with the purpose of improving or enabling participation in different roles, habits, routines, and rituals in the home, school, workplace, and community, among other settings (43). Additionally, an intervention plan that systematically considers patient demands related to clinical and functional findings, performance in ADLs and occupational roles, as constructed in the present study, must be formulated for symptomatic patients with HAM.

Living with HTLV is complex and results in radical changes, which often impact the daily lives of people with HAM. The changes are highly correlated with the symptoms of the disease and require special care regarding daily activities, FC and skills that require more effort from the lower limbs and posture and balance transfers (44).

Thus, occupational therapists can develop interventions and strategies, such as environmental adaptations, appropriate wheelchair use and ADL training, thus fostering greater independence and helping improve the quality of life of these individuals.

Professional occupational therapists use their knowledge about the relationship between individuals, their involvement in meaningful occupations and their context to design intervention plans based on occupations that facilitate changes or improvements in patient factors (values, beliefs and spirituality; body functions; and body structures) and performance skills (motor skills, procedural, and social interactions) necessary for successful participation (43). These professionals seek results through patient participation and thus facilitate involvement through adaptations and modifications in the environment or to local objects when necessary (33).

Occupational therapy services are provided for rehabilitation and promotion of the health and well-being of individuals with disabilities or with needs unrelated to disability. These services include the acquisition and preservation of occupational identity for those who have or are at risk of developing disease, injury, illness, disorders, conditions, impairment, disability, activity limitations, or participation restrictions (43).

In this particular study, the importance of understanding changes in occupational roles due to illness is revealed, as this demand has been studied by occupational therapy to support clinical practice in the creation of strategies to recover occupational roles and autonomy.

In addition, professionals must be trained to provide correct guidance to patients (44) and family members and to encourage such patients to establish a support network (45). Such measures can create a favorable environment for adherence to a rehabilitation program (35). This study sought to strengthen other perspectives on how health professionals can intervene with patients infected with HTLV, including broadening the understanding of occupational dimensions.

People living with HAM suffer from physical manifestations and symptoms that limit their social participation (46). In this context, occupational therapy in the care of patients infected with HTLV-1 can contribute to reducing the difficulties encountered by these patients when adapting to a new routine, changing occupational roles and learning and developing life skills with the maximum possible autonomy (44).

Occupational therapists recognize that people’s health is maintained when they are able to engage in occupations performed at home, at work and in the community. Furthermore, these professionals are also concerned with the variety of factors that hinder or strengthen people’s involvement and participation in occupations (43). Thus, the impacts of the COVID-19 pandemic on the occupational life of PLHTLV must be considered, including repercussions for the mental health of these subjects and the consequences of the infection for the FC of people with HAM who already have limitations, such as ADL performance and social interaction constraints related to HAM. Notably, further studies should examine the implications of the COVID-19 pandemic for the occupations of PLHTLV, especially those with HAM symptoms, and target knowledge acquisition that can support the interventions of occupational therapists during the pandemic and postpandemic period.

## Conclusion

The limitation of the study was the inability to associate patient performance with the time of infection at which symptoms emerge. The impact of HAM on the ADLs of PLHTLV can be reduced through the use of an intervention plan that includes occupational therapists as part of a multidisciplinary treatment team in rehabilitation programs, representing an important third-level prevention initiative that supports short-, medium-, and long-term goals depending on the therapeutic needs of patients. Asymptomatic individuals infected with HTLV-1 should be monitored to prevent future losses in ADLs and occupational roles.

Interventions by health professionals should consider all dimensions of each human being, including occupations, and occupational therapists are responsible for involving functionality and occupational roles, among other domains, in their actions and strategies to promote patient autonomy.

## Data Availability Statement

The original contributions presented in the study are included in the article/Supplementary Material, further inquiries can be directed to the corresponding author.

## Ethics Statement

The studies involving human participants were reviewed and approved by Research Ethics Committee of the Health Sciences Institute (Instituto de Ciências da Saúde – ICS) of the Federal University of Pará (Universidade Federal do Pará – UFPA). The patients/participants provided their written informed consent to participate in this study.

## Author Contributions

ES, CA-A, AV, and IC: study or research design, and analysis and interpretation of data. DP, AV, and IC: final manuscript review with critical and intellectual participation. All authors contributed to the article and approved the submitted version.

## Conflict of Interest

The authors declare that the research was conducted in the absence of any commercial or financial relationships that could be construed as a potential conflict of interest.

## Publisher’s Note

All claims expressed in this article are solely those of the authors and do not necessarily represent those of their affiliated organizations, or those of the publisher, the editors and the reviewers. Any product that may be evaluated in this article, or claim that may be made by its manufacturer, is not guaranteed or endorsed by the publisher.
