# Supplementary material for: Occupational Therapy for People Living With Human T-Lymphotropic Virus 1: Importance and Effectiveness of an Intervention Plan at the Level of Prevention and Rehabilitation
Source: Front Med (Lausanne). 2022 Mar 29;9:859889. doi: 10.3389/fmed.2022.859889 (PMC9001934; doi:10.3389/fmed.2022.859889)
Supplement: Supplementary file 1 [file Table_1.pdf]

Supplementary Material - Identification of occupational problems and proposed occupational therapy intervention plan for symptomatic patients.

DEFINITIONS OF OCCUPATIONAL THERAPY DOMAINS (31)	IDENTIFICATION OF OCCUPATIONAL PROBLEMS (31)	OCCUPATIONAL THERAPY INTERVENTIONS (31)	TARGET RESULTS (31)
**Patient factors:**<br>The patient can be a person, people, group (collective of individuals, for example, families) or population.<br>Patient factors are specific characteristic abilities or beliefs that are part of a person and that influence occupational performance. Patient factors include values, beliefs and spirituality; body functions; and body structure.	(i) Impaired body functions:<br>- Physiological functions of human systems<br>- Sensory functions: presence of diffuse pain in the lumbar region and lower limbs; and presence of occasional and permanent paresthesias, especially in the lower limbs<br>- Neuromusculoskeletal and movement-related functions: presence of spasticity, marked by spasms (involuntary contraction) of flexor/extensor muscle groups in the upper and/or lower limbs; altered gait patterns, with disability or difficulty in performing ADLs, more specifically functional mobility, for example, chair-to-bed transfers	• Sensory and neuromusculoskeletal functions related to movement:<br>- Provide a structured sensory environment in an outpatient clinic and at home (for example, through movement or tactile sensations using materials of different textures for stimulation)<br>- Perform exercises to strengthen the upper and lower limbs oriented toward ADLs, using therapeutic weight and elastic bands, among other resources<br>- Develop gait training, taking into account the limitations of the patient and the environment.<br>- Use integrative health practices such as Pilates and acupuncture for pain relief and general improvements in sensorimotor symptoms.	• Sensory and neuromusculoskeletal functions related to movement:<br>Patients should observe a decrease and report less discomfort in relation to symptoms such as paresthesia and spasms, improving the sensory and motor responses, based on the demands of the environment.
**Occupational performance skills:**	(ii) impaired motor skills:	• Motor skills:	• Motor skills:

Actions aimed at observable goals such as small units of involvement in occupations. They are learned and developed across time and are situated in specific contexts and environments. They are characterized as motor skills, process skills and social interaction skills.	- Observed while the person interacts with and moves objects and moves in an environment in which the task is performed<br>- Walking (walking and running): Inability or difficulty walking on flat surfaces, due to poor balance, instability, lack of support or use of inadequate mobility devices during the execution of tasks<br>- Inability or difficulty completing tasks because of physical fatigue and needing to take a break to recover<br>- Loss of balance or difficulty balancing when standing or jumping with both feet or 1 foot	- Use muscle relaxation techniques<br>- Provide guidance on the use of energy conservation techniques when performing occupational tasks<br>- Provide guidance on the use of appropriate positioning when performing ADLs<br>- Provide instructions on how to operate an adequate mobility device and verify financial and use conditions	The patient should report less attrition before, during and after the performance of occupational tasks, observe a decrease in postural instability, preventing events such as falls, and recognize the best use of mobility aids.
**Patterns of occupational performance:** Habits, routines, roles and rituals used in the process of engaging in occupations or activities; these patterns can support or hinder occupational performance.	(iii) Changes in occupational roles:<br>- Set of behaviors expected by society, modeled by culture and determined by context that can be further conceptualized and defined by the patient<br>- Negative changes in the performance of the worker role linked to paid formal or informal work and negative changes in the home related to housework (management of the home); negative changes in the role of	• Occupational roles:<br>- Collaborate with the patient and family to establish necessary routines so that they can resume roles or improve the effectiveness in the most significant and important roles for the patient	• Occupational roles:<br>The patient must have the ability to recognize that he or she effectively meets the demands of roles in which he or she is involved and signal the recovery of previously performed roles.

	friends, evidenced by withdrawal or difficulty in forming new bonds in the community, compromising social participation		
**Occupation:** Occupation indicates everything that people want, need or should do, whether physical, mental, social, sexual, political or spiritual, including rest and sleep. It refers to all the real aspects of doing, of being and becoming human and of belonging (Wilcock and Townsend, 2014).<br><br>Occupations are classified into<br><br>ADLs, IADLs, rest and sleep, education, work, play, leisure, and social participation.	(iv) changes in the performance of ADLs, IADLs, and work and in social participation:<br><br>• ADLs (activities oriented toward self-care):<br><br>- Urination: impaired urinary elimination (change in bladder control)<br><br>-Functional mobility: difficulty moving from one position or place to another (when performing daily activities, for example, chair-to-bed transfers and climbing up and down stairs)<br><br>• IADLs (activities that support daily life at home and in the community that often require more complex interactions than those used in ADLs):<br><br>- Establishment and management of the home: inability or difficulty in	• ADLs:<br><br>- Urination:<br><br>- Establish monitoring of urinary elimination (urination) with the patient and family and promote the recording of frequency, consistency, odor, volume and color, as appropriate<br><br>- Educate the patient and his or her family about the signs and symptoms of urinary tract infection<br><br>- Guide the patient in developing a routine for using the toilet<br><br>- Functional mobility:<br><br>- Increase the functional mobility of the patient in the home environment, with an emphasis on transfers, including functional ambulation and the transport of objects<br><br>• IADLs:<br><br>- Management of the home:<br>- Analyze the physical environment required for activities (e.g., size, arrangement, surface, lighting, temperature, noise, humidity, and ventilation)	• ADLs:<br><br>The patient should make progress through reductions in occasional accidents related to urination and better performance in functional mobility.<br><br>• IADLs:<br><br>The patient will recognize the importance of changes in the home environment through better performance of occupational tasks, with the absence of risk events, such as falls, and making appropriate use of resources and equipment for better home and health management, thus increasing well-being and quality of life<br><br>• Work:<br><br>The patient will engage in desired occupations in ways that lead to personal satisfaction and are congruent with the expectations within that culture, recognizing limitations and skills that enhance the productive being.<br><br>• Social participation:

	obtaining and maintaining personal, household and environmental goods, including the maintenance and repair of personal goods (clothing and household items) and knowing how to seek help or whom to contact<br><br>- Health management and maintenance: inability or difficulty to develop, manage and maintain health routines and promote well-being, such as physical fitness and a reduction in health risk behaviors (use of condoms, for example)<br><br>• Work (occupations that are performed with or without financial reward)<br><br>- Difficulty in determining skills, developing interests and skills, selecting appropriate non-professional activities and adjusting lifestyle in the absence of the worker role<br><br>• Social participation (interrelationship of occupations to support the desired involvement in community and family activities as well as those involving peers and friends	- Offer guidelines and establish goals by mutual agreement on possible changes in the home environment to promote safety and comfort when performing activities<br><br>- Health management and maintenance:<br>- Assist the patient in the development of routines for health promotion and disease prevention<br>- Assist the patient in the management and maintenance of healthy habits (such as condom use)<br>- Provide real information regarding diagnosis, treatment and prognosis<br>- Guide the use of devices and provide resources that help establish a routine for health promotion<br>• Work:<br><br>- Assist the patient in the recognition of skills/aptitudes that can be used to stimulate productivity<br>- Promote the development of interests and discovery of skills for the selection of appropriate non-professional activities and make lifestyle adjustments to account for the absence of the worker role, for example, voluntary activities<br>• Social participation:<br><br>- Develop group interventions in the community and outpatient clinics, among other environments, based on collective demands, thus allowing clients to explore and develop skills for social participation, including basic social interaction skills, self-regulation tools, and goal-setting and decision-making strategies	The patient will expand and strengthen their support network.

	-Family members: inability or difficulty engaging in activities that result in successful interactions in specific, necessary and/or desired family roles<br><br>- Friends: inability or difficulty engaging in activities with different levels of interaction and intimacy with friends		
